# Metafounders are related to *F*_st_ fixation indices and reduce bias in single-step genomic evaluations

**DOI:** 10.1186/s12711-017-0309-2

**Published:** 2017-03-10

**Authors:** Carolina A. Garcia-Baccino, Andres Legarra, Ole F. Christensen, Ignacy Misztal, Ivan Pocrnic, Zulma G. Vitezica, Rodolfo J. C. Cantet

**Affiliations:** 1 Departamento de Producción Animal, Facultad de Agronomía, Universidad de Buenos Aires, C1417DSE Buenos Aires, Argentina; 2 Instituto de Investigaciones en Producción Animal - Consejo Nacional de Investigaciones Científicas y Técnicas, Buenos Aires, Argentina; 3 GenPhySE, INRA, INPT, ENVT, Université de Toulouse, 31326 Castanet-Tolosan, France; 4 Center for Quantitative Genetics and Genomics, Department of Molecular Biology and Genetics, Aarhus University, 8830 Tjele, Denmark; 5 Animal and Dairy Science, University of Georgia, Athens, GA 30602 USA

## Abstract

**Background:**

Metafounders are pseudo-individuals that encapsulate genetic heterozygosity and relationships within and across base pedigree populations, i.e. ancestral populations. This work addresses the estimation and usefulness of metafounder relationships in single-step genomic best linear unbiased prediction (ssGBLUP).

**Results:**

We show that ancestral relationship parameters are proportional to standardized covariances of base allelic frequencies across populations, such as $F_{\text{st}}$ fixation indexes. These covariances of base allelic frequencies can be estimated from marker genotypes of related recent individuals and pedigree. Simple methods for their estimation include naïve computation of allele frequencies from marker genotypes or a method of moments that equates average pedigree-based and marker-based relationships. Complex methods include generalized least squares (best linear unbiased estimator (BLUE)) or maximum likelihood based on pedigree relationships. To our knowledge, methods to infer $F_{\text{st}}$ coefficients from marker data have not been developed for related individuals. We derived a genomic relationship matrix, compatible with pedigree relationships, that is constructed as a cross-product of {−1,0,1} codes and that is equivalent (apart from scale factors) to an identity-by-state relationship matrix at genome-wide markers. Using a simulation with a single population under selection in which only males and youngest animals are genotyped, we observed that generalized least squares or maximum likelihood gave accurate and unbiased estimates of the ancestral relationship parameter (true value: 0.40) whereas the naïve method and the method of moments were biased (average estimates of 0.43 and 0.35). We also observed that genomic evaluation by ssGBLUP using metafounders was less biased in terms of estimates of genetic trend (bias of 0.01 instead of 0.12), resulted in less overdispersed (0.94 instead of 0.99) and as accurate (0.74) estimates of breeding values than ssGBLUP without metafounders and provided consistent estimates of heritability.

**Conclusions:**

Estimation of metafounder relationships can be achieved using BLUP-like methods with pedigree and markers. Inclusion of metafounder relationships reduces bias of genomic predictions with no loss in accuracy.

## Background

Metafounders are pseudo-individuals that describe relationships within and across pedigree base populations. The concept of metafounders provides a coherent framework for the theory of genomic evaluation [1]. Genomic evaluation in agricultural species often implies partially genotyped populations, i.e. some individuals are genotyped, using high-density genetic markers across the genome, others are not, and phenotypes may be recorded in either of the two subsets. An integrated solution called single-step genomic best linear unbiased prediction (ssGBLUP) has been proposed [2–4]. This solution uses the following integrated relationship matrix:

$${\mathbf{H}} = \left( {\begin{array}{*{20}c} {{\mathbf{A}}_{11} - {\mathbf{A}}_{12} {\mathbf{A}}_{22}^{ - 1} {\mathbf{A}}_{21} + {\mathbf{A}}_{12} {\mathbf{A}}_{22}^{ - 1} {\mathbf{GA}}_{22}^{ - 1} {\mathbf{A}}_{21} } & {\quad {\mathbf{A}}_{12} {\mathbf{A}}_{22}^{ - 1} {\mathbf{G}}} \\ {{\mathbf{GA}}_{22}^{ - 1} {\mathbf{A}}_{21} } & {\quad {\mathbf{G}}} \\ \end{array} } \right),$$

with inverse:

$${\mathbf{H}}^{ - 1} = {\mathbf{A}}^{ - 1} + \left( {\begin{array}{*{20}c} {\mathbf{0}} & {\quad {\mathbf{0}}} \\ {\mathbf{0}} & {\quad {\mathbf{G}}^{ - 1} - {\mathbf{A}}_{22}^{ - 1} } \\ \end{array} } \right),$$

where ${\mathbf{G}}$ is the genomic relationship matrix, ${\mathbf{A}}$ is the pedigree-based relationship matrix, and matrices ${\mathbf{A}}_{11} ,{\mathbf{A}}_{12} ,{\mathbf{A}}_{21} ,{\mathbf{A}}_{22}$ are submatrices of ${\mathbf{A}}$ with labels 1 and 2 denoting non-genotyped and genotyped individuals, respectively.

Because genotyped animals are often not a random sample from the analyzed populations (they tend to be younger or selected), it was quickly acknowledged that a proper analysis requires specifying different means for genotyped and non-genotyped individuals for the trait under consideration. These different means can be considered as parameters of the model, which are either fixed [4] or random [5, 6] effects. In the latter case, the random variables induce covariances between individuals, a situation that is informally referred to as “compatibility” of genomic and pedigree relationships. In fact, compatibility implies equality of the average breeding value of the base population and of the genetic variance [7] across the different measures of relationships. Numerically, the problem appears as follows. The formulae for matrix ${\mathbf{H}}$ and its inverse contain (${\mathbf{G}} - {\mathbf{A}}_{22}$) and (${\mathbf{G}}^{ - 1} - {\mathbf{A}}_{22}^{ - 1}$) (assuming ${\mathbf{G}}$ is full rank), respectively. This suggests that if ${\mathbf{G}}$ and ${\mathbf{A}}_{22}$ differ too much, biases may appear.

Genomic relationships are usually computed in one of two manners: using “cross-products” [8] or “corrected identity-by-state (IBS)” [9]. Both depend critically on assumed allele frequencies at markers in the pedigree base population [10]. Base allele frequencies are often unavailable. However, for most purposes, allele frequencies are not of interest *per se* and can be treated as nuisance parameters that can be marginalized. Christensen [11] achieved an algebraic integration of allele frequencies, leading to a very simple covariance structure with allele frequencies in genomic relationships fixed at 0.5 (e.g., using genotypes coded as {−1,0,1} in the cross-product method) and a parameter called $\gamma$ that describes relationships between pedigree founders i.e. ${\mathbf{A}}_{base}^{{\left( {{\upgamma }} \right)}} = {\mathbf{I}}\left( {1 - \frac{{{\upgamma }}}{2}} \right) + {\mathbf{11}}^{\prime } {{\upgamma }}$ in the base population. A second parameter in Christensen’s marginalisation is $s$, which is a measure of marker heterozygosity in the base population. Therefore, instead of inferring (thousands of) base allele frequencies, inference can be based on two simple parameters $\gamma$ and $s$. Both can be estimated by maximizing the likelihood of observed genotypes. In addition, this approach considers the fact that pedigree depth is arbitrary and mostly based on historical availability of records.

Legarra et al. [1] showed the equivalence of the Christensen approach to the metafounder concept: pseudo-individuals that encapsulate three ideas: (a) separate means for each base population [4, 12, 13], (b) randomness of these means [5] and (c) propagation of the randomness of these means to the progeny [11], while accommodating several populations with complex crosses, e.g. [14]. Legarra et al. [1] also generalized one relationship between founders (scalar $\gamma$) to several relationships between founders in the pedigree, i.e. ancestral relationships (matrix ${\varvec{\Gamma}}$), and suggested simple methods to estimate them. Legarra et al. [1] showed that construction of ${\mathbf{A}}^{{\varvec{\Gamma}}}$ from ${\varvec{\Gamma}}$ and a pedigree reduces to the use of the tabular rules [15] for construction of relationships, and its inversion is achieved by inversion of ${\varvec{\Gamma}}$ followed by Henderson’s rules [16]. We provide an example of matrices ${\mathbf{A}}^{{\varvec{\Gamma}}}$ and ${\varvec{\Gamma}}$ in “Appendix”. However, the performance of their model has not been tested so far, either for estimation of ancestral relationships or for genomic evaluation.

This work has two objectives. The first is to show that the structure of the metafounder approach yields an alternative parameterization and method for estimation of ancestral relationships. By doing so, we found that ancestral relationships are generalizations of Wright’s $F_{\text{st}}$ fixation index [17]. The second goal is to test, by simulation, (1) methods to estimate ancestral relationship parameters, (2) the quality of genomic predictions using metafounders, and (3) the quality of variance component estimation. For the second goal, the simulated population is undergoing selection and with a complete partially genotyped pedigree.

## Methods

### Relationship between metafounders and allele frequencies in the pedigree base population

#### Single population

 Let ${\mathbf{M}}$ be a matrix of genotypes coded as gene content, i.e. {0,1,2} and the genomic relationship matrix ${\mathbf{G}} = \left( {{\mathbf{M}} - {\mathbf{J}}} \right)\left( {{\mathbf{M}} - {\mathbf{J}}} \right)^{\prime } /s,$ with ${\mathbf{J}}$ a matrix of 1s, with reference alleles taken at random, so that the expected allele frequency $p$ is 0.5 for a random locus [11]. In other words, the matrix ${\mathbf{Z}} = \left( {{\mathbf{M}} - {\mathbf{J}}} \right)$ contains values of {−1,0,1} for each genotype. In a single population, let $\gamma$ be the relationship coefficient between pedigree founders or, equivalently, the self-relationship of the metafounder [1, 11]. Parameter $s$ (defined above) is a measure of marker heterozygosity in the population. Ancestral relationships in $\gamma$ explain, for instance, genomic relationships in ${\mathbf{G}} = \left( {{\mathbf{M}} - {\mathbf{J}}} \right)\left( {{\mathbf{M}} - {\mathbf{J}}} \right)^{\prime } /s$ that are not captured by available pedigree; e.g. across nominally unrelated individuals. It will be shown later that this relationship $\gamma$ is relative to a population with maximum heterozygosity and is analogous to an $F_{\text{st}}$ fixation index [18].

Christensen [11] estimated the two parameters, $\gamma$ and $s$, using maximum likelihood, whereas [1] suggested methods of moments. Closer inspection of Appendix A in [11] leads to the following developments that were not described in Christensen [11] (see “Appendix” of the present paper for more details).

Parameter $\gamma$ is such that $\gamma = \frac{{4Var\left( {p_{i} } \right)}}{{2Var\left( {p_{i} } \right) + E\left( {2p_{i} q_{i} } \right)}}$, with $p_{i} = 1 - q_{i}$ the allele frequency at a random locus $i$. Parameter $s = n\left( {2Var\left( {p_{i} } \right) + E\left( {2p_{i} q_{i} } \right)} \right)$, with $n$ being the number of markers. However, $E\left( {2p_{i} q_{i} } \right) = 2E\left( {p_{i} } \right)E\left( {q_{i} } \right) - 2Var\left( {p_{i} } \right) = 0.5 - 2Var\left( {p_{i} } \right),$ such that if reference alleles are chosen at random across loci, then $E\left( {p_{i} } \right) = E\left( {q_{i} } \right) = 0.5$. From this it follows that:

$$s = \frac{n}{2} = \frac{number\;of\;markers}{2},$$

and the genomic relationship matrix is ${\mathbf{G}} = 2\left( {{\mathbf{M}} - {\mathbf{J}}} \right)\left( {{\mathbf{M}} - {\mathbf{J}}} \right)^{\prime } /n$. Interestingly, this matrix is similar to a matrix of IBS relationships, that can be written as:

$${\mathbf{G}}_{IBS} = \left( {{\mathbf{M}} - {\mathbf{J}}} \right)\left( {{\mathbf{M}} - {\mathbf{J}}} \right)^{'} /n + {\mathbf{11}}^{\prime } ,$$

so that ${\mathbf{G}}_{IBS} = \frac{1}{2}{\mathbf{G}} + {\mathbf{11}}^{\prime }$ (see proof in “Appendix”).

Substituting $E\left( {2p_{i} q_{i} } \right) = 0.5 - 2Var\left( {p_{i} } \right)$ into the expression $\gamma = \frac{{4Var\left( {p_{i} } \right)}}{{2Var\left( {p_{i} } \right) + E\left( {2p_{i} q_{i} } \right)}}$ gives: $\gamma = 8 Var\left( {p_{i} } \right) = 8\sigma_{p}^{2}$, such that $\gamma$ for a single population is eight times the variance of allele frequencies in the base population (this variance was described by Cockerham [19]). We stress that $Var\left( {p_{i} } \right) = \sigma_{p}^{2}$ to imply that $\sigma_{p}^{2}$ (and $\gamma$) is a parameter, the variance of allele frequencies across markers [10, 11, 20, 21]. However, $s$ can be considered as equivalent to heterozygosity when all markers have an allele frequency of 0.5, that is, the maximum possible heterozygosity.

#### Multiple populations

In an analogous manner, the relationship between two metafounders $b$ and $b^{\prime}$ is: $\gamma_{{b,b^{\prime } }} = 8Cov\left( {p_{b,i} ,p_{{b^{\prime},i}} } \right) = 8\sigma_{{p_{b} ,p_{{b^{\prime } }} }}$, i.e., the covariance across loci between allele frequencies of two populations $b$ and $b^{\prime }$. This is almost tautological: the relationship (between two populations in this case) is the covariance between the gene content at a locus. Christensen et al. [6] implicitly show this in Appendix A of their paper. Cockerham [19] and Robertson [22] interpreted 4 $\sigma_{{p_{b} ,p_{{b^{\prime } }} }}$ as the coancestry between two populations and Fariello et al. [23] used $\sigma_{{p_{b} ,p_{{b^{\prime } }} }}$ to describe the divergence of populations. Several measures of genetic distance between populations have been developed (e.g. [24]), and most of them contain a term that is related, implicitly or explicitly, to $\sigma_{{p_{b} ,p_{{b^{\prime } }} }}$. In particular, the average square of the Euclidean distance can be written as $D^{2} = E\left( {\left( {p_{b} - p_{{b^{\prime}}} } \right)^{2} } \right) = - 2\sigma_{{p_{b} ,p_{{b^{\prime } }} }}$. Thus, $\gamma_{{b,b^{\prime } }} = - 4D^{2}$.

### Estimation

#### Estimation in a single population

Estimation of $s$ is trivial, it is simply half the number of markers. Parameter $\gamma$ is proportional to the variance of allele frequencies in the base population. If base population individuals were genotyped, computing allele frequencies and estimating $\gamma$ would be trivial. In the next section, we propose methods when this is not the case, i.e. genotyped individuals are related and perhaps several generations away from the base population.

*Assuming no pedigree structure i.e. naïve* The simplest model assumes that genotyped individuals are unrelated and constitute the base population. For locus $i$, let $\varvec{m}_{i}$ be a vector of gene contents in the form {0,1,2}, defined as before. The mean of this vector is $\mu_{i} = 2p_{i} .$ For each locus, $\mu_{i}$ is estimated as the observed mean of $\varvec{m}_{i}$, then $Var\left( {\hat{\varvec{\mu }}} \right)$ is computed as the empirical variance across loci of $\hat{\varvec{\mu }} = \left( {\hat{\mu}_{1} , \ldots ,\hat{\mu }_{n} } \right)$, and because $p_{i} = \mu_{i} /2$, then $\hat{\sigma }_{p}^{2} = Var\left( {\hat{\varvec{\mu }}} \right)/4$ and $\gamma = 8\hat{\sigma }_{p}^{2} = 2Var\left( {\hat{\varvec{\mu }}} \right)$.

*Considering pedigree structure* At locus $$i$$, gene content can be seen as a quantitative trait mean of $$\varvec{m}_{\varvec{i}}$$ in the base population equal to $$2p_{i}$$, where $$p_{i}$$ is the allele frequency in the base population and the genetic variance is $$2p_{i} q_{i}$$ [25–27]. Cockerham [19] showed that the covariance of gene content of marker $$i$$ between individuals $$j$$ and $$k$$ is a function of their relationship ($$A_{jk} )$$: $$Cov\left( {m_{i,j} ,m_{i,k} } \right) = A_{jk} 2p_{i} q_{i}$$. A linear model can therefore be written as:

$${\mathbf{m}}_{i} = {\mathbf{1}}\mu_{i} + {\mathbf{Wu}}_{i} + {\mathbf{e}},$$

where ${\mathbf{W}}$ is an incidence matrix relating individuals in the pedigree to observed genotypes, and ${\mathbf{u}}_{i}$ is the deviation of each individual from the mean $\mu_{i}$ for all individuals [25–27]. Assuming multivariate normality: ${\varvec{\upmu}}\sim N\left( {{\mathbf{0}},{\mathbf{I}}\sigma_{\mu }^{2} } \right)$ and ${\mathbf{u}}_{i} \sim N\left( {{\mathbf{0}},{\mathbf{A}}\left( {2p_{i} q_{i} } \right) } \right) = N\left( {{\mathbf{0}},{\mathbf{A}}\sigma_{{m_{i} }}^{2} } \right)$.

Equivalently, for the set of genotyped individuals (labelled as “2”), ${\mathbf{u}}_{2,i} \sim N\left( {{\mathbf{0}},{\mathbf{A}}_{22} \left( {2p_{i} q_{i} } \right) } \right)$, where ${\mathbf{A}}_{22} = {\mathbf{WAW}}^{\prime }$ is an additive relationship matrix that includes only the genotyped individuals. From this formulation, there are two possible strategies to estimate $\sigma_{\mu }^{2}$.

*Generalized least squares (GLS)* This ignores the prior distribution of $\varvec{ }{\varvec{\upmu}}$ and estimates each $\mu_{i}$ as a “fixed effect”, using best linear unbiased estimator (BLUE) (or, equivalently, GLS) estimators of $\mu_{i}$ separately for each locus. One option is to use the ${\mathbf{A}}^{ - 1}$ spanning all the pedigree and mixed model equations [25–27]. Equivalently, the corresponding GLS expression is:

$$\hat{\mu }_{i} = \left( {{\mathbf{1}}^{\prime } {\mathbf{A}}_{22}^{ - 1} \sigma_{{m_{i} }}^{ - 2} {\mathbf{1}}} \right)^{ - 1} {\mathbf{1}}^{\prime } {\mathbf{A}}_{22}^{ - 1} \varvec{m}_{i} \sigma_{{m_{i} }}^{ - 2} = \left( {{\mathbf{1}}^{\prime } {\mathbf{A}}_{22}^{ - 1} {\mathbf{1}}} \right)^{ - 1} {\mathbf{1}}^{\prime } {\mathbf{A}}_{22}^{ - 1} \varvec{m}_{i} ,$$

where $\left( {{\mathbf{1}}^{\prime } {\mathbf{A}}_{22}^{ - 1} {\mathbf{1}}} \right)$ is the sum of elements of ${\mathbf{A}}_{22}^{ - 1}$, $\sigma_{{m_{i} }}^{2} = 2p_{i} q_{i}$ and ${\mathbf{1}}^{\prime } {\mathbf{A}}_{22}^{ - 1} \varvec{m}_{i}$ is a weighted sum of genotypes. Then, $\sigma_{\mu }^{2}$ is estimated as $Var\left( {{\hat{\varvec{\upmu }}}} \right)$, because $p_{i} = \mu_{i} /2$, $\hat{\sigma }_{p}^{2} = \sigma_{\mu }^{2} /4$, and it follows that $\hat{\gamma } = 2\hat{\sigma }_{\mu }^{2}$.

*Maximum likelihood* If allele frequencies in the base population have a distribution, $\mu_{i}$ can be considered as drawn from a normal distribution, ${\varvec{\upmu}}\sim N\left( {{\mathbf{0,I}}\sigma_{\mu }^{2} } \right)$. Thus $\sigma_{\mu }^{2}$ is a variance component that can be estimated by maximum likelihood (ML). The equations for given values of $\sigma_{\mu }^{2}$ and $\sigma_{{m_{i} }}^{2} = 2p_{i} q_{i}$ are $\left( {{\mathbf{1}}^{{\prime }} {\mathbf{A}}_{22}^{ - 1} \sigma_{{m_{i} }}^{ - 2} {\mathbf{1}} + \sigma_{\mu }^{ - 2} } \right)\hat{\mu }_{i} = {\mathbf{1}}^{\prime } {\mathbf{A}}_{22}^{ - 1} \sigma_{{m_{i} }}^{ - 2} {\mathbf{m}}_{i}$. An expectation–maximization scheme [28] to obtain ML is as follows. Pick starting values for $\sigma_{\mu }^{2}$ and $\sigma_{{m_{i} }}^{2}$. Iterate until convergence on:

1. For each marker $i$,
  - estimate $$\hat{\mu }_{i} = \left( {{\mathbf{1}}^{\prime } {\mathbf{A}}_{22}^{ - 1} \sigma_{{m_{i} }}^{ - 2} {\mathbf{1}} + \sigma_{\mu }^{ - 2} } \right)^{ - 1} {\mathbf{1}}^{\prime } {\mathbf{A}}_{22}^{ - 1} \sigma_{{m_{i} }}^{ - 2} {\mathbf{m}}_{i}$$,
  - store $$PEV_{i} \left( {\hat{\mu }_{i} } \right) = \left( {\sigma_{\mu }^{ - 2} + {\mathbf{1}}^{\prime } {\mathbf{A}}_{22}^{ - 1} \sigma_{{m_{i} }}^{ - 2} {\mathbf{1}}} \right)^{ - 1}$$,
  - update $\sigma_{{m_{i} }}^{2}$ as $\hat{\sigma }_{{m_{i} }}^{2} = 2\hat{p}_{i} \hat{q}_{i}$ with $\hat{p}_{i} = \hat{\mu }_{i} /2$;
2. Update $\sigma_{\mu }^{2}$ as $\hat{\sigma }_{\mu }^{2} = \frac{1}{n}\left( {\varvec{ }{\hat{\varvec{\upmu }}}^{\prime } {\hat{\varvec{\upmu }}} + \sum PEV_{i} \left( {\hat{\mu }_{i} } \right)} \right)$, where the second part of the expression corresponds to the trace $tr\left( {{\mathbf{IC}}} \right)$, ${\mathbf{I}}$, the identity matrix, is the relationship structure across levels of $\varvec{ }{\varvec{\upmu}}$ and ${\mathbf{C}}$ is the prediction error covariance matrix of ${\hat{\varvec{\upmu }}}$. As only the diagonal elements of ${\mathbf{C}}$ are needed in $tr\left( {{\mathbf{IC}}} \right)$, its elements $PEV_{i} \left( {\hat{\mu }_{i} } \right)$ can be obtained separately from each single locus analysis.

At convergence, the estimate is $\hat{\gamma } = 2\hat{\sigma }_{\mu }^{2}$. This gives the same estimate as the method based on a Wishart likelihood function [11] with $s = n/2$ (results not shown).

### Estimation in multiple populations

If $t$ base populations are considered, the variance component $\sigma_{\mu }^{2}$ generalizes to ${\varvec{\Sigma}}_{0}$, a $t \times t$ matrix of variances and covariances between means $\mu_{i}^{\left[ b \right]}$ for marker $i$ in population $b$. Across populations, $${\varvec{\Sigma}}_{0} = \left( {\begin{array}{*{20}c} {\sigma_{{\mu^{\left[ 1 \right]} \mu^{\left[ 1 \right]} }}^{2} } & {\quad \sigma_{{\mu^{\left[ 1 \right]} \mu^{\left[ 2 \right]} }} } & {\quad \ldots } \\ \ldots & {\quad \sigma_{{\mu^{\left[ 2 \right]} \mu^{\left[ 2 \right]} }}^{2} } & {\quad \ldots } \\ \ldots & {\quad \ldots } & {\quad \ldots } \\ \end{array} } \right)$$ and ${\hat{\varvec{\Gamma }}} = 2{\hat{\varvec{\Sigma }}}_{0}$.

#### Assuming no pedigree structure

*Naïve* If relationships across individuals are ignored:

$${\mathbf{m}}_{i} = {\mathbf{Q}}{\varvec{\upmu}}_{i} + {\mathbf{e}}_{i} ,$$

where ${\mathbf{Q}}$ is a matrix, the rows of which sum to 1, and that assigns individuals to fractions of populations, and ${\varvec{\upmu}}_{i}$ is a vector with $t$ elements for the average of each population. For each locus, ${\varvec{\upmu}}_{i}$ can be estimated using least squares and the covariance matrix of ${\varvec{\upmu}}_{i}$ across loci gives an estimate of ${\varvec{\Sigma}}_{0}$, e.g. for two populations ${\hat{\varvec{\Sigma }}}_{0} = Cov\left( {{\varvec{\upmu}}^{\left[ 1 \right]} ,{\varvec{\upmu}}^{\left[ 2 \right]} } \right)$, a two-by-two matrix.

#### Considering pedigree structure

If there are no crosses between individuals from different populations in the pedigree, the estimation of allele frequencies in each base population can be split in separate analyses by population $b$: ${\mathbf{m}}_{i}^{b} = {\mathbf{1}}\mu_{i}^{\left[ b \right]} + {\mathbf{W}}^{b} {\mathbf{u}}_{i}^{b} + {\mathbf{e}}$, with ${\mathbf{u}}_{i}^{b} \sim N\left( {{\mathbf{0}},{\mathbf{A}}^{b} \left( {2p_{i} \left( {1 - p_{i} } \right)} \right)} \right)$ and ${\mathbf{A}}^{b}$ the matrix of pedigree-based relationships among individuals in population *b*, and the analysis proceeds as in a single population. Then, ${\hat{\varvec{\Sigma }}}_{0}$ is estimated as the observed matrix of covariances for $\hat{\mu }_{i}^{b}$ across loci.

When there are crosses, there are two alternatives.

#### Generalized least squares (GLS)

The first alternative [27] is to use a genetic groups model [12, 13], as ${\mathbf{m}}_{i} = {\mathbf{Q}}{\varvec{\upmu}}_{i} + {\mathbf{Wu}}_{i} + {\mathbf{e}}$, where ${\mathbf{Q}}_{k,b}$ contains the fraction of ancestry $b$ in individual $k$. This ignores the fact that the variance of gene content, $\left( {2p_{i} q_{i} } \right)$, differs between breeds and crosses. The second, and more exact alternative, is to use the representation where the breeding values are split into within- and across-breed components [29]:

$${\mathbf{m}}_{i} = {\mathbf{Q}}{\varvec{\upmu}}_{i} + \mathop \sum \limits_{b} {\mathbf{W}}^{b} {\mathbf{u}}_{i}^{b} + \mathop \sum \limits_{{b,b^{\prime } ,b > b^{\prime } }} {\mathbf{W}}^{{b,b^{\prime } }} {\mathbf{u}}_{i}^{{b,b^{\prime } }} + {\mathbf{e}},$$

with partial relationship matrices for vectors ${\mathbf{u}}_{i}^{b}$ and ${\mathbf{u}}_{i}^{{b,b^{\prime } }}$. The BLUE’s of ${\varvec{\upmu}}_{i}$ can be obtained and then ${\hat{\varvec{\Sigma }}}_{0}$ estimated as above.

#### Maximum likelihood (ML)

Analogously to the single population case, an expectation–maximization updated estimate can be obtained using multiple-trait formulations [28], where $PEC$ is the prediction error variance–covariance, e.g. with two populations:

$${\varvec{\Sigma}}_{0} = \left( {\begin{array}{*{20}c} {{\varvec{\upmu}}^{{\left[ 1 \right]^{\prime } }} {\varvec{\upmu}}^{\left[ 1 \right]} } & {\quad {\varvec{\upmu}}^{{\left[ 1 \right]^{\prime } }} {\varvec{\upmu}}^{\left[ 2 \right]} } \\ {\varvec{\upmu}^{{\left[ 2 \right]^{\prime } }}\varvec{\upmu}^{\left[ 1 \right]} } & {\quad\varvec{\upmu}^{{\left[ 2 \right]^{\prime } }}\varvec{\upmu}^{\left[ 2 \right]} } \\ \end{array} } \right).$$

Our implementation of this approach is as follows:

1. For each marker $i$:
  - estimate $${\hat{\varvec{\upmu }}}_{i} = \left( {{\varvec{\Sigma}}_{0}^{ - 1} + {\mathbf{Q}}^{\prime } {\mathbf{A}}_{22}^{ - 1} \sigma_{{m_{i} }}^{ - 2} {\mathbf{Q}}} \right)^{ - 1} {\mathbf{Q}}^{\prime } {\mathbf{A}}_{22}^{ - 1} \sigma_{{m_{i} }}^{ - 2} {\mathbf{m}}_{i}$$,
  - store $$PEC_{i} \left( {{\hat{\varvec{\upmu }}}_{i} } \right) = \left( {{\varvec{\Sigma}}_{0}^{ - 1} + {\mathbf{Q}}^{\prime } {\mathbf{A}}_{22}^{ - 1} \sigma_{{m_{i} }}^{ - 2} {\mathbf{Q}}} \right)^{ - 1}$$,
  - update $\sigma_{{m_{i} }}^{2}$ as $\hat{\sigma }_{{m_{i} }}^{2} = 2\hat{p}_{i}^{*} \left( {1 - \hat{p}_{i}^{*} } \right)$ with $\hat{p}_{i}^{*} = \frac{1}{Nb}\mathop \sum \nolimits_{b = 1,Nb} \frac{{\hat{\mu }_{i}^{b} }}{2}$;
2. Update ${\varvec{\Sigma}}_{0}$ using cross-products within and across populations as, e.g., with two populations:

$${\hat{\varvec{\Sigma }}}_{0} = \frac{1}{n}\left( {\left( {\begin{array}{*{20}c} {{\hat{\varvec{\upmu }}}^{{\left[ 1 \right]^{{\prime }} }} {\hat{\varvec{\upmu }}}^{\left[ 1 \right]} } & {\quad {\hat{\varvec{\upmu }}}^{{\left[ 1 \right]^{\prime } }} {\hat{\varvec{\upmu }}}^{\left[ 2 \right]} } \\ {{\hat{\varvec{\upmu }}}^{{\left[ 2 \right]^{{\prime }} }} {\hat{\varvec{\upmu }}}^{\left[ 1 \right]} } & {\quad {\hat{\varvec{\upmu }}}^{{\left[ 2 \right]^{\prime } }} {\hat{\varvec{\upmu }}}^{\left[ 2 \right]} } \\ \end{array} } \right) + \mathop \sum \limits_{i = 1,n} PEC_{i} } \right).$$

Step 1 includes an approximation in (1c) because we assume that $\sigma_{{m_{i} }}^{2} = 2p_{i} q_{i}$ is the same for all base populations, as in the GLS above, which could be further improved by using partial relationship matrices. This point will be addressed in future research.

### Simulation

To assess the quality of genomic predictions using one metafounder, we simulated data using QMSim [30]. The simulation closely followed that in [5] to mimic a dairy cattle selection scheme scenario. A historical population undergoing mutation and drift was generated, followed by a recent population undergoing selection.

First, 100 generations of the historical population were generated with an effective population size of 100 during the first 95 generations, followed by a gradual expansion during the last five generations to an effective population size of 3000. Thirty chromosomes of 100 cM and 40,000 segregating biallelic markers distributed at random along the chromosomes in the first generation of the historical population were simulated. The 40,000 markers were resampled from a larger set of 90,000 markers in order to obtain allelic frequencies from a beta(2,2) distribution, similar to dairy cattle marker data, so that parameter $\gamma$ had a true value around 0.40. There were 1500 QTL affecting the phenotype; QTL allele effects were sampled from a Gamma distribution with a shape parameter of 0.4. Mutation rate at the markers (recurrent mutation process) and QTL was assumed to be 2.5 × 10^−5^ per locus per generation [31]. We used a higher mutation rate than typical (10^−8^, [32, 33]) to overcome the fact that QMSim is not a coalescent simulator. Phenotypes for a trait recorded only on females with a heritability of 0.30 were simulated.

Then, 10 overlapping generations of selection followed. In each generation, 200 males were mated with 2600 females to produce 2600 offspring by a positive assortative mating design based on EBV. Within the simulation, individuals were selected according to estimated breeding value (EBV) based on pedigree BLUP. In each generation, 40% of males and 20% of females were replaced by selected younger individuals. No restrictions were set to avoid or minimize inbreeding, so highly inbred individuals were found, as a result of strong selection and matings among highly-related individuals. A total of 100 individuals had an inbreeding coefficient higher than 0.20 (mainly found in the last generation), with some individuals having inbreeding coefficients higher than 0.40. True breeding values (TBV) and pedigree information were available for all 10 generations (28,800 individuals in the pedigree), phenotypes were available for all females except in the last generation (14,300 records). The 840 sires of females with phenotypic records were genotyped, as well as 2600 individuals in generation 9 (with records) and 2600 in generation 10 (without records). A total of 20 independent replicates were made. A two-step analysis was carried out using the simulated data. First, we compared several methods to estimate $\gamma$. Then, we tested the quality of genomic predictions using four methods (see section on genomic prediction methods), one of which included one metafounder.

### Methods to estimate ${\varvec{\upgamma}}$

Parameter $\gamma$ was estimated using four estimation methods. First, the naïve method that does not consider the pedigree structure. Pedigree information was included in three methods: GLS, ML, and the method of moments (MM) in [1]. For a single population, the last method involves estimation of $\gamma$ based on summary statistics of ${\mathbf{A}}_{22}$ (regular pedigree-relationship matrix for genotyped individuals) and ${\mathbf{G}}$ (the genomic relationship matrix).

### Genomic prediction methods

The EBV of the selection candidates in generation 10 (genotyped and without phenotype records) were estimated using four methods. The first was the pedigree-based BLUP (PBLUP) based on phenotype and pedigree information. The second method was ssGBLUP, in which genomic information is also taken into account. We used the correction of [34] to equate genomic and pedigree average inbreeding and relationships, the default method used in most practical applications [34, 35]. However, the implementation that we used does not include inbreeding in the setup of ${\mathbf{A}}^{ - 1}$ [36], although it does consider inbreeding in ${\mathbf{A}}_{22}^{ - 1}$ (see below for use of these matrices). The third method was ssGBLUP that includes inbreeding in the setup of ${\mathbf{A}}^{ - 1}$ and of ${\mathbf{A}}_{22}^{ - 1}$ (ssGBLUP_F). The fourth method was ssGBLUP with the metafounder (ssGBLUP_M), using $\gamma$ estimated by GLS since it turned out to be an accurate method to estimate ${\varvec{\Gamma}}$ (see the Results section). The four methods used the following inverse relationship matrices: PBLUP: ${\mathbf{A}}^{ - 1}$; ssGBLUP: $${\mathbf{H}}^{ - 1} = {\mathbf{A}}^{ - 1} + \left( {\begin{array}{*{20}c} 0 & {\quad 0} \\ 0 & {\quad {\mathbf{G}}_{a}^{ - 1} - {\mathbf{A}}_{22}^{ - 1} } \\ \end{array} } \right)$$ where ${\mathbf{G}}_{a}$ is as in [34] and ${\mathbf{A}}^{ - 1}$ is constructed ignoring inbreeding [36]; ssGBLUP_F: same as ssGBLUP, with ${\mathbf{A}}^{ - 1}$ correctly constructed; ssGBLUP_M: $${\mathbf{H}}^{\left( \gamma \right) - 1} = {\mathbf{A}}^{\left( \gamma \right) - 1} + \left( {\begin{array}{*{20}c} 0 & {\quad 0} \\ 0 & {\quad {\mathbf{G}}^{ - 1} - {\mathbf{A}}_{22}^{\left( \gamma \right) - 1} } \\ \end{array} } \right)$$ where ${\mathbf{G}} = \left( {{\mathbf{M}} - {\mathbf{J}}} \right)\left( {{\mathbf{M}} - {\mathbf{J}}} \right)^{\prime } /s$ with $s = n/2$ (see the "Methods" section) and ${\mathbf{A}}^{\left( \gamma \right)}$ is as in [1]. More details are given in “Appendix”. For computation, we used blupf90 [37]. In the case of ssGBLUP_M, we constructed ${\mathbf{H}}^{\left( \gamma \right) - 1}$ with own software and then used the option user_file in blupf90 (http://nce.ads.uga.edu/wiki/doku.php).

### Quality of genomic prediction

Prediction quality was evaluated for all 2600 selection candidates in generation 10. The accuracy of the methods was measured as the Pearson correlation between TBV and EBV. Bias was calculated as the difference between the average TBV and average EBV with respect to the base population (i.e. to the solution of the metafounder for ssGBLUP_M or to 0 for the other methods). Thus, bias is related to estimated genetic progress in the selection candidates. The inflation (often also called bias) of the prediction method was quantified by the coefficient of regression of TBV on EBV. These two statistics correspond to the coefficients $b_{0}$ and $b_{1}$ in the Interbull validation method [38], which uses the regression $TBV = b_{0} + b_{1} EBV + e$. The mean square error (MSE) of prediction of EBV was calculated as the mean of the squared difference between TBV and EBV. An ideal method should have maximum accuracy, minimum MSE, zero bias, and a regression coefficient of 1. These are not only elegant statistical properties but also have relevance in livestock selection [39–41]. Changes in ranking of the selection candidates were also assessed by calculating the Spearman’s rank correlation coefficients between EBV across methods.

In addition, the quality of variance component estimation was assessed by comparing estimated and simulated heritabilities. For this purpose, variance components were estimated by REML with remlf90 [37] based on the four methods (PBLUP, ssGBLUP, ssGBLUP_F, ssGBLUP_M).

## Results

### Estimation of ${\varvec{\upgamma}}$

**Fig. 1:**
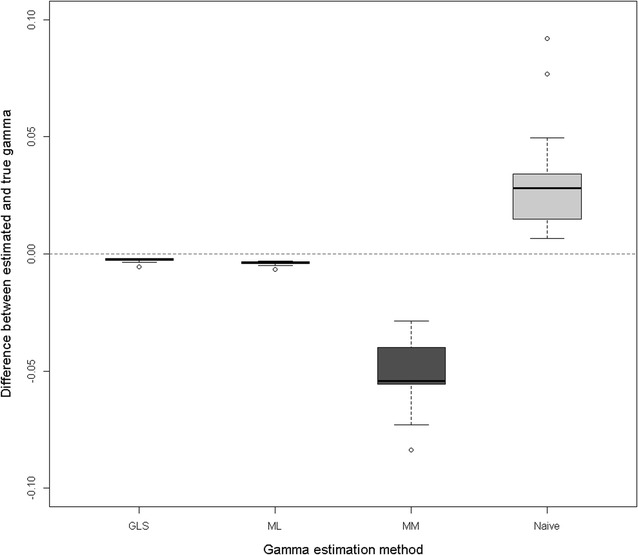
Differences between estimated and true Gamma, across 20 simulation replicates. Gamma was estimated by generalized least squares (GLS), maximum likelihood (ML), method of moments (MM) and the Naive method

Figure 1 shows boxplots of the differences between the estimates of $\gamma$ based on the four methods (MM, Naïve, ML and GLS) and the true values obtained by simulation, for each of the 20 replicates. The simulations were tailored to produce $\gamma = 0.40$. Methods ML and GLS estimated $\gamma$ very accurately. Method MM clearly underestimated $\gamma$, whereas the Naïve method overestimated it. Based on these results, we used $\gamma$ estimated by GLS for ssGBLUP_M for prediction. The effect of employing different values of $\gamma$ in genomic prediction was assessed to quantify its impact on predictions. Using estimates of $\gamma$ based on MM only slightly changed results. For example, the accuracies and slopes of ssGBLUP_M were not affected up to the 4th digit (not shown).

### Quality of genomic prediction

**Fig. 2:**
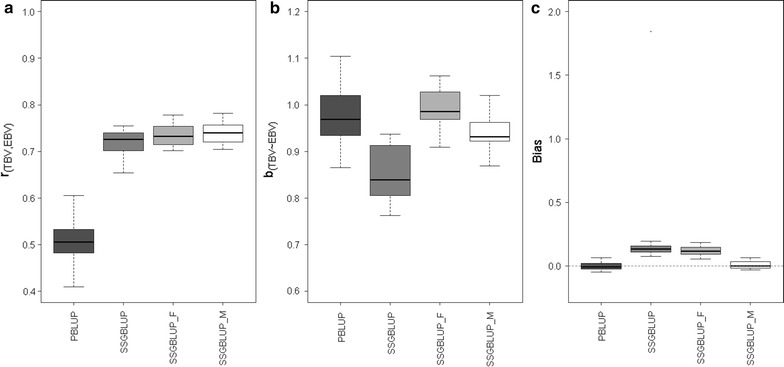
**a** Correlation of TBV with EVB for each prediction method (accuracy). **b** Regression slope of TBV on EBV (overdispersion). **c** Bias [average (EBV–TBV)]

Correlations between TBV and EBV of candidates in generation 10 for each prediction methods are in Table 1 and Fig. 2a. Compared with PBLUP, ssGBLUP_F and ssGBLUP_M increased accuracy by approximately 23 absolute points. This shows an important improvement by including marker information in the prediction and the possibility of generating a small extra gain when also including the metafounder. Method ssGBLUP resulted in a small loss of accuracy compared to ssGBLUP_F and ssGBLUP_M.

**Table 1:** Accuracy (correlation between TBV and EBV), inflation (regression coefficient of TBV on EBV), bias [average (EBV–TBV)] and mean square error (MSE) for each prediction methods

Prediction method	Accuracy	Inflation	Bias	MSE
PBLUP	0.51 (0.05)	0.98 (0.06)	−0.0003 (0.03)	0.206 (0.01)
ssGBLUP	0.72 (0.03)	0.89 (0.19)	0.2169 (0.04)	0.159 (0.03)
ssGBLUP_F	0.74 (0.02)	0.99 (0.04)	0.1167 (0.04)	0.141 (0.01)
ssGBLUP_M	0.74 (0.02)	0.94 (0.04)	0.0094 (0.03)	0.125 (0.01)

Averages across 20 replicates with standard deviations in parenthesis

Table 1 and Fig. 2b display the regression coefficient of TBV on EBV, which measures the degree of inflation for each prediction method and should be close to 1. PBLUP and ssGBLUP_F produced values closest to 1. Including genomic data in the prediction based on ssGBLUP resulted in regression coefficients lower than 1, but including the metafounder in ssGBLUP_M gave values closer to 1. Methods ssGBLUP_M and ssGBLUP_F displayed a lower standard deviation compared to the other two methods. Again, method ssGBLUP showed the highest variability.

Biases of EBV obtained with each prediction method are in Table 1 and Fig. 2c. Both PBLUP and ssGBLUP_M were unbiased, whereas ssGBLUP and ssGBLUP_F were biased. The bias was higher for ssGBLUP than for ssGBLUP_F, which was largely due to a single outlier; the median bias was roughly the same for ssGBLUP and ssGBLUP_F. The bias with ssGBLUP_F was equivalent to roughly 0.5 generations of genetic improvement or 0.4 standard genetic deviations. Finally, ssGBLUP_M had the lowest MSE (closer to zero), followed by ssGBLUP_F (Table 1).

### Ranking of EBV

The methods were also compared based on rank correlations of EBV with TBV and between methods. A rank correlation of 1 implies that the same candidates would be selected. Results are in Table 2. Rank correlations with TBV were similar to the Pearson correlations in Table 1. Selection decisions differed only slightly when using ssGBLUP, ssGBLUP_F or ssGBLUP_M. Note, however, that this table reports rank correlations among young selection candidates in the last generation and does not address comparisons across generations (e.g. old vs. young animals), which is sensitive to the biases that are reflected in Table 1 [41]. For instance, all young animals would be overestimated by 0.11 with ssGBLUP_F, which results in these young animals looking better than proven sires, which had an accuracy of essentially 1 and no bias. Depending on the selection scheme, this may lead to less than optimal selection decisions.

**Table 2:** Spearman correlations among TBV and the four EBV for each prediction methods

	EBV PBLUP	EBV ssGBLUP	EBV ssGBLUP_F	EBV ssGBLUP_M
TBV	0.49 (0.06)	0.71 (0.02)	0.72 (0.03)	0.73 (0.02)
EBV PBLUP		0.56 (0.05)	0.62 (0.04)	0.64 (0.04)
EBV ssGBLUP			0.99 (0.01)	0.98 (0.01)
EBV ssGBLUP_F				0.99 (0.002)

Averages across 20 replicates with standard deviations in parenthesis

### Estimation of variance components

**Fig. 3:**
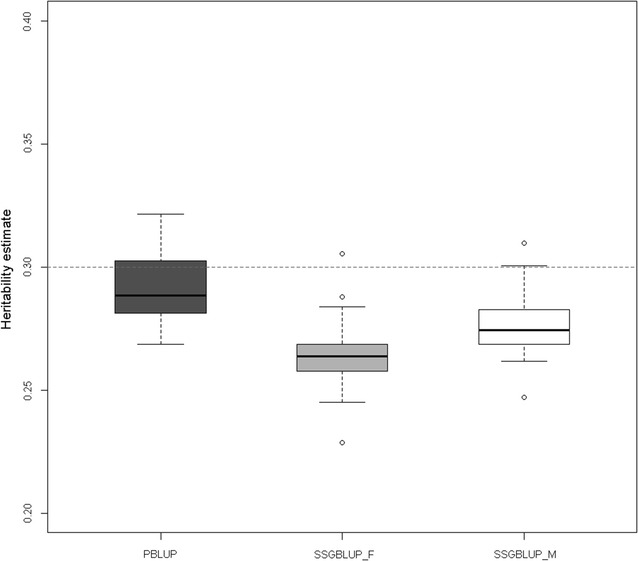
Estimated heritability for PBLUP, SSGBLUP_F and SSGBLUP_M considering the 20 replicates. The *dotted line* shows the simulated heritability of 0.30

Figure 3 shows estimates of heritability obtained with three of the four methods (PBLUP, ssGBLUP_F and ssGBLUP_M). The estimates obtained using ssGBLUP did not converge for six of the 20 simulation replicates. Convergence was achieved in those cases by weighting the submatrix ${\mathbf{A}}_{22}^{ - 1}$ in ${\mathbf{H}}^{ - 1}$ by $\omega = 0.7$ instead of 1 [42] but poor quality estimates were obtained and are, therefore, not reported.

Estimates were generally lower than the simulated true heritability (0.30). The lowest estimates were obtained with ssGBLUP_F. Including the metafounder improved estimates compared to ssGBLUP_F and reduced variability of estimates compared to PBLUP.

## Discussion

In this work, we have addressed the complex issue of conciliation of marker and pedigree information in genetic evaluation. Powell et al. [43] argued that both IBS (at the markers) and identity-by-descent (IBD) are compatible notions because they are both measures of identity at causal genes. However, incompatibility appears when mixing both types of relationships [5, 34, 44, 45]. Legarra [7] suggested that, in order to compare genetic variance across IBD, IBS or other measures of relationships, a common reference must be chosen. Similar (but not identical) to [43], in this work we used a fixed reference (${\mathbf{G}}$ constructed as a cross-product of {−1,0,1} genotypic codes) and tailored ${\mathbf{A}}$ (IBD, pedigree) to fit ${\mathbf{G}}$ (IBS, markers). Compared to previous approaches, using a fixed reference has the advantage that genomic relationships are immutable (i.e. adding more genotyped individuals to the database does not change the existing relationships) and they do not depend on pedigree depth, which by construction is always limited and, in animal breeding, often heterogeneous. Our approach is in fact very similar to using IBS as measure of identity. We used a genomic relationship matrix ${\mathbf{G}} = 2\left( {{\mathbf{M}} - {\mathbf{J}}} \right)\left( {{\mathbf{M}} - {\mathbf{J}}} \right)^{\prime } /n$, whereas the matrix of IBS is ${\mathbf{G}}_{IBS} = {\mathbf{G}}/2 + {\mathbf{11}}^{{\prime }}$ (see proof in “Appendix”). In GBLUP with associated variance component estimation, when all animals are genotyped, using a model with ${\mathbf{G}}_{IBS}$ or the ${\mathbf{G}}$ matrix proposed here yields identical EBV, as the term ½ in ${\mathbf{G}}/2$ gets absorbed into the variance component and the constant ${\mathbf{11}}^{{\prime }}$ gets absorbed into the fixed part of the linear mixed model [7, 46]. However, matrix ${\mathbf{G}}$ rather than ${\mathbf{G}}_{IBS}$ must be used in ssGBLUP_M because ${\mathbf{G}}_{IBS}$ is not compatible with pedigree relationships. In [4], the (fixed effect) intercept term $\mu_{g}$ models, identical to [5], the difference between genetic values of individuals in the base and genotyped individuals. These intercept terms play therefore a similar role as metafounders.

### Easy estimation of ancestral relationships

Derivations in the Theory section show that estimation of ancestral relationships based on $\gamma$ (one base population) and ${\varvec{\Gamma}}$ (several base populations) can be framed within the classic linear model approach of quantitative genetics [19], which has recently been used for gene content [14, 25–27]. This approach is easy to understand and compute. Also, ${\varvec{\Gamma}}$ can be understood, just like heritability, as an unobserved base population parameter that does not change with additional data (although its estimate may change). Therefore, an accurate estimate of ${\varvec{\Gamma}}$ can be used repeatedly without the need for re-estimation, as is customary in livestock genetic evaluation. This contrasts with “centering” of marker covariates, which changes with every new genotype. If all base allele frequencies were known exactly, then there should be no need to use metafounders, as relationship matrices can be appropriately constructed [14].

In the work presented here, the simplest methods (Naïve and method of moments) yielded biased (upwards and downwards, respectively) estimates of $\gamma$; the naïve method because it ignores that allele frequencies tend to drift to their extreme values as generations progress, and the method of moments because it implicitly assumes that genotyped individuals are a random sample from a particular generation.

Equivalence of ancestral relationships with second moments of allele frequencies also shows a strong relation with population genetics theory, which will be detailed in the next paragraph.

### Relationship between metafounders parameter ${\varvec{\upgamma}}$ and $\varvec{F}_{{{\mathbf{st}}}}$ fixation index

The fixation index $F_{\text{st}}$ [17] is a measure of diversity of a set of populations with respect to a reference population, usually the pool of all populations. In this approach, each population is assumed to be a random sample from all possible populations that could be sampled according to the evolutionary process described by $F_{\text{st}}$. Conceptually, $F_{\text{st}}$ is a parameter to be estimated [18, 19], and it is not a statistic computed from the data. The usual definition of $F_{\text{st}}$ for a particular biallelic locus is:

$$F_{\text{st}} = \frac{{\sigma_{p}^{2} }}{{\bar{p}\left( {1 - \bar{p}} \right)}},$$

where $\sigma_{p}^{2}$ is the variance of allelic frequencies across populations and $\bar{p}$ is the allele frequency of the conceptual combined population. If we consider that the variance of allele frequencies applies *across* loci and not *across* populations, it follows that $\bar{p} = 0.5$ because reference alleles are taken at random. In this case:

$$F_{\text{st}} = \frac{{\sigma_{p}^{2} }}{{\bar{p}\left( {1 - \bar{p}} \right)}} = \frac{{\sigma_{p}^{2} }}{{0.5^{2} }} = 4\sigma_{p}^{2} = \frac{\gamma }{2}.$$

Our interpretation of this link between $F_{\text{st}}$ and $\gamma$ is as follows. Jacquard [47] called $\frac{\gamma }{2}$ the “inbreeding coefficient of a population”. Cockerham [16] modelled $\frac{\gamma }{2} = \theta_{l} = F_{\text{st}}$ as an intraclass correlation, “the coancestry of the line with itself”, in other words, the probability that two gametes taken at random from the population are identical. Thus, it makes perfect sense to consider that the additive relationship (which is twice the coancestry value) of a group with itself is $\gamma = 2\theta_{l} = 8\sigma_{p}^{2}$. This is the interpretation of the $\frac{\gamma }{2}$ coefficient in Legarra et al. [1]. Note that the assumption that $\bar{p} = 0.5$ is automatically fulfilled if reference alleles are chosen at random across loci (i.e., they are neither the most frequent nor the least observed allele).

Alternatively, [1] showed that for a population with self-relationships equal to $\gamma$, the average heterozygosity is $1 - \frac{\gamma }{2}$, i.e. the variance is reduced by an amount equal to $\frac{\gamma }{2}$ from the conceptual population with heterozygosity 1. Thus $\frac{\gamma }{2}$ can be interpreted as $F_{\text{st}}$ if the $F_{\text{st}}$ is taken as a measure of homozygosity.

### Consequences of using metafounders in genomic evaluation

Genomic estimates of breeding values are invariant to allele coding [46] when all individuals are genotyped. However, this is not the case when pedigree and marker informations are combined, as in ssGBLUP. In this work, we have shown that, even in the presence of complete pedigree and a single base population, use of metafounders in ssGBLUP_M leads to slightly more inflated and less biased EBV, lower MSE, and nearly unbiased estimates of heritability compared to ssGBLUP_F. Bias, defined as E(EBV–TBV), is typically overlooked in genomic prediction, but in an example of biased evaluation, Henderson [48] recognized that “sires of later generations appeared to be under-evaluated relative to older sires”. Overdispersion, also called bias in recent literature (e.g. [38]), may also have a dramatic impact in practice [39–41] and the trade-off between bias and variance needs further study. For instance, Vitezica et al. [5] found that ssGBLUP_F was unbiased but had some overdispersion, which likely depends on the data structure, including which individuals are genotyped.

In addition, use of metafounders allows a clear definition of genomic relationships because relationships do not depend on pedigree depth or completeness or on changes in allele frequencies as new data is added. In addition, a high-dimensional parameter (i.e. base allele frequencies) is substituted by a low-dimensional one (matrix ${\varvec{\Gamma}}$).

The poor performance of ssGBLUP compared to ssGBLUP_F is likely due to the presence of highly inbred individuals because ssGBLUP ignored inbreeding in the setup of ${\mathbf{A}}^{ - 1}$. This relates to the interpretation of parameter $\omega$, as used in early studies of ssGBLUP [42]. An application of ssGBLUP for type traits in Holstein [42] experienced convergence problems, which were eliminated when ${\mathbf{A}}_{22}^{ - 1}$ was multiplied by $\omega = 0.7$ and which increased accuracy of predictions. However, the nature of parameter $\omega$ was not known [49]. In those studies, the inverse of the numerator relationship matrix ${\mathbf{A}}^{ - 1}$ was constructed using Henderson’s rules while ignoring inbreeding [36], while the submatrix ${\mathbf{A}}_{22}^{ - 1}$ included inbreeding. As a result, the elements in the latter matrix were too large. In addition, genotyped animals were on average unrelated in ${\mathbf{G}}$ but not in ${\mathbf{A}}_{22}$, which can be corrected by scaling ${\mathbf{G}}$, as in [5]. However, this resulted in the elements in ${\mathbf{A}}_{22}^{ - 1}$ to be too large for younger animals relative to ${\mathbf{G}}$. Both these problems are partially circumvented by putting a weight $\omega < 1$ on ${\mathbf{A}}_{22}^{ - 1}$. When ${\mathbf{A}}^{ - 1}$ was constructed while considering inbreeding, the optimal value of $\omega$ in an analysis of Holstein dairy cattle increased from 0.7 to 0.9 (Masuda, personal communication, 2016). However, the metafounder approach provides a more principled solution to this problem. Also, following these experiences, ${\mathbf{A}}^{ - 1}$ should always be constructed while considering inbreeding to avoid infrequent but pathological problems.

## Conclusions

Metafounders have relationships that are closely related to $F_{\text{st}}$ fixation indices and proportional to covariances of allele frequencies in base populations. Use of metafounders can be simplified by new methods to estimate the covariance of base allele frequencies. We verified by simulation of a selected population that, in a single population, both GLS and ML are unbiased and computationally efficient. In the same simulation, use of metafounders in ssGBLUP led to more accurate and less biased evaluations, and also to more accurate estimates of genetic parameters. We propose a genomic relationship matrix that refers to a population with ideal base allele frequencies equal to 0.5. This matrix is similar to an IBS relationship matrix (up to scale factors), does not change with new data, and is compatible with pedigree data if metafounders are used. In the simulated data, pedigrees were perfectly known. Future work with real datasets in more complex settings—purebreds and their crosses [50, 51], and selected populations with unknown parent groups [13] will investigate the feasibility and accuracy, in practice, of using metafounders in ssGBLUP.

## Appendix

The appendix contains examples, details and algebraic developments that were not detailed in the main text.

### Example of relationship matrix with one metafounder

Consider the pedigree:

$$\begin{array}{*{20}c} 1 & {\quad 0} & 0 \\ 2 & {\quad 1} & {\quad 1} \\ 3 & {\quad 1} & {\quad 1} \\ 4 & {\quad 2} & {\quad 3} \\ 5 & {\quad 2} & {\quad 4} \\ \end{array}$$

where 1 is a metafounder with $\gamma = a_{1,1} = 0.2$. Using the tabular method [15], $a_{2,2} = 1 + a_{1,1} /2 = 1.1$ and $a_{1,2} = 0.5\left( {a_{1,sire\left( 2 \right)} + a_{1,dam\left( 2 \right)} } \right) = 0.5\left( {a_{1,1} + a_{1,1} } \right) = 0.2.$ Proceeding with the tabular method, ${\mathbf{A}}^{\left( \gamma \right)}$ is:

$$\begin{array}{*{20}c} {0.2000} & {\quad 0.2000} & {\quad 0.2000} & {\quad 0.2000} & {\quad 0.2000} \\ {0.2000} & {\quad 1.1000} & {\quad 0.2000} & {\quad 0.6500} & {\quad 0.8750} \\ {0.2000} & {\quad 0.2000} & {\quad 1.1000} & {\quad 0.6500} & {\quad 0.4250} \\ {0.2000} & {\quad 0.6500} & {\quad 0.6500} & {\quad 1.1000} & {\quad 0.8750} \\ {0.2000} & {\quad 0.8750} & {\quad 0.4250} & {\quad 0.8750} & {\quad 1.3250} \\ \end{array}$$

with inverse ${\mathbf{A}}^{\left( \gamma \right) - 1}$, that can be obtained by inverting $\gamma$ and using Henderson’s rules [1, 16]:

$$\begin{array}{*{20}c} {7.2222} & {\quad - 1.1111} & {\quad - 1.1111} & {\quad 0.0000} & {\quad 0.0000} \\ { - 1.1111} & {\quad 2.2222} & {\quad 0.5556} & {\quad - 0.5556} & {\quad - 1.1111} \\ { - 1.1111} & {\quad 0.5556} & {\quad 1.6667} & {\quad - 1.1111} & {\quad 0.0000} \\ {0.0000} & {\quad - 0.5556} & {\quad - 1.1111} & {\quad 2.7778} & {\quad - 1.1111} \\ {0.0000} & {\quad - 1.1111} & {\quad 0.0000} & {\quad - 1.1111} & {\quad 2.2222} \\ \end{array}$$

These compare with regular ${\mathbf{A}}$ that can be obtained by setting $\gamma = 0$. In this case, individual 1 is an unknown parent group and its “relationships” have been set to 0 for presentation:

$$\begin{array}{*{20}c} {0.0000} & {\quad 0.0000} & {\quad 0.0000} & {\quad 0.0000} & {\quad 0.0000} \\ {0.0000} & {\quad 1.0000} & {\quad 0.0000} & {\quad 0.5000} & {\quad 0.7500} \\ {0.0000} & {\quad 0.0000} & {\quad 1.0000} & {\quad 0.5000} & {\quad 0.2500} \\ {0.0000} & {\quad 0.5000} & {\quad 0.5000} & {\quad 1.0000} & {\quad 0.7500} \\ {0.0000} & {\quad 0.7500} & {\quad 0.2500} & {\quad 0.7500} & {\quad 1.2500} \\ \end{array}$$

and the inverse relationship matrix including the unknown parent group [13] is ${\mathbf{A}}^{ - 1}$:

$$\begin{array}{*{20}c} {2.0000} & {\quad - 1.0000} & {\quad - 1.0000} & {\quad 0.0000} & {\quad 0.0000} \\ { - 1.0000} & {\quad 2.0000} & {\quad 0.5000} & {\quad - 0.5000} & {\quad - 1.0000} \\ { - 1.0000} & {\quad 0.5000} & {\quad 1.5000} & {\quad - 1.0000} & {\quad 0.0000} \\ {0.0000} & {\quad - 0.5000} & {\quad - 1.0000} & {\quad 2.5000} & {\quad - 1.0000} \\ {0.0000} & {\quad - 1.0000} & {\quad 0.0000} & {\quad - 1.0000} & {\quad 2.0000} \\ \end{array}$$

### Analytical derivation of $\varvec{\gamma}$ and $\varvec{s}$

For a particular population, the genetic variance–covariance structure is a function of two parameters $\eta_{1}$ and $\eta_{2}$: $\gamma = \frac{{4\eta_{1} }}{{2\eta_{1} + \eta_{2} }}$ and $s = n\left( {2\eta_{1} + \eta_{2} } \right)$ ($n$ being the number of markers) which depend on the allelic frequencies Appendix A in [11]. With $p_{j}$ being the allelic frequencies across the $j = 1 \ldots n$ loci, these parameters do not depend on $j$ and are equal to:

$$\eta_{1} = Var\left( {p_{j} } \right),$$

$$\eta_{2} = E\left( {2p_{j} q_{j} } \right),$$

with $$q = 1 - p$$.

Now, we use the following developments.

1 $$E\left( {pq} \right) = E\left( {p\left( {1 - p} \right)} \right) = E\left( p \right) - E\left( {p^{2} } \right).$$

Since we have $Var\left( p \right) = E\left( {p^{2} } \right) - E\left( p \right)^{2}$, we obtain $E\left( {p^{2} } \right) = Var\left( p \right) + E\left( p \right)^{2}$. We also have $E\left( q \right) = 1 - E\left( p \right)$. Substituting $E\left( {p^{2} } \right)$ in Eq. (1) gives:

$$\begin{aligned} E\left( {pq} \right) & = E\left( p \right) - Var\left( p \right) - E\left( p \right)^{2} \\ & = E\left( p \right)\left( {1 - E\left( p \right)} \right) - Var\left( p \right) = E\left( p \right)E\left( q \right) - Var\left( p \right). \\ \end{aligned}$$

If markers are biallelic and labeled at random $E\left( p \right) = E\left( q \right) = 0.5$. So the equation above gives $E\left( {pq} \right) = 0.25 - Var\left( p \right)$. From this we obtain:

$$2\eta_{1} + \eta_{2} = 2Var\left( {p_{j} } \right) + 0.5 - 2Var\left( {p_{j} } \right) = 0.5,$$

and therefore

2 $$s = n \left( {2\eta_{1} + \eta_{2} } \right) = \frac{n}{2},$$

or, in other words, $s$ is half the number of markers. Furthermore,

3 $$\gamma = \frac{{4\eta_{1} }}{{2\eta_{1} + \eta_{2} }} = \frac{{4\eta_{1} }}{0.5} = 8 Var\left( {p_{j} } \right) = 8\sigma_{p}^{2} ,$$

so that $\gamma$ for a single population is eight times the variance of allelic frequencies at the base population.

### Equivalences of genomic relationship matrices

The matrix ${\mathbf{G}}$ described in [11] and in this paper can be written as:

$${\mathbf{G}} = \frac{2}{n}\left( {{\mathbf{M}} - {\mathbf{J}}} \right)\left( {{\mathbf{M}} - {\mathbf{J}}} \right)^{\prime } ,$$

where ${\mathbf{M}}$ contains genotypes coded as {0,1,2} and ${\mathbf{J}}$ is a matrix of 1s. The purpose of this paragraph is to show the linear relationship of this matrix with a matrix describing IBS coefficients, in fact ${\mathbf{G}}_{IBS} = \frac{1}{2}{\mathbf{G}} + {\mathbf{11}}^{\prime }$. The terms in ${\mathbf{G}}_{IBS}$ are usually described in terms of identities or countings (i.e. [9, 10, 52]):

$$G_{{IBS_{ij} }} = \frac{1}{n}\mathop \sum \limits_{m = 1}^{n} 2 \frac{{\mathop \sum \nolimits_{k = 1}^{2} \mathop \sum \nolimits_{l = 1}^{2} I_{kl} }}{4},$$

where $I_{kl}$ measures the identity (with value 1 or 0) of allele $k$ in individual $i$ with allele $l$ in individual $j$, and single-locus identity measures are averaged across $k$ loci.There is an algebraic expression for this “counting”. Toro et al. [10] in their Eq. (1) show that, for biallelic markers, for a locus $k$ (omitted in the notation for clarity):

4 $$f_{{M_{ij} }} = \frac{{m_{i} }}{2}\frac{{m_{j} }}{2} + \left( {1 - \frac{{m_{i} }}{2}} \right)\left( {1 - \frac{{m_{j} }}{2}} \right),$$

for coancestry (half relationship) $f_{{M_{ij} }}$ of individuals $i$ and $j$, where $m/2$ is the “gene frequency” of the individual (half the gene content ($m$), i.e. {0,1/2,1} for the three genotypes).

In order to prove ${\mathbf{G}}_{IBS} = \frac{1}{2}{\mathbf{G}} + {\mathbf{11}}^{\prime }$, first we translate the equation in [10] to the more familiar scale of relationships $g_{IBS_{ij}} = 2f_{{M_{ij} }}$ and gene contents $m$. Thus:

$$\begin{aligned} g_{{IBS_{ij} }} & = 2f_{{M_{ij} }} = 2\left( {\frac{{m_{i} }}{2}\frac{{m_{j} }}{2} + \left( {\frac{2}{2} - \frac{{m_{i} }}{2}} \right)\left( {\frac{2}{2} - \frac{{m_{j} }}{2}} \right)} \right) \\ g_{{IBS_{ij} }} & = m_{i} m_{j} - m_{i} - m_{j} + 2. \\ \end{aligned}$$

This expression can be easily verified in a table with the nine possible genotypes:

**Table:**

	AA	Aa	aa
AA	2	1	0
Aa	1	1	1
aa	0	1	2

Also,

$$g_{IBS_{ij}} = m_{i} m_{j} - m_{i} - m_{j} + 2 = \left( {m_{i} - 1} \right)\left( {m_{j} - 1} \right) + 1,$$

which extends to all individuals and averaged across loci can be written as:

$${\mathbf{G}}_{IBS} = \frac{1}{n}\left( {{\mathbf{M}} - {\mathbf{J}}} \right)\left( {{\mathbf{M}} - {\mathbf{J}}} \right)^{\prime } + {\mathbf{11}}^{\prime } .$$

Thus, matrix $\varvec{G}_{IBS} = \frac{1}{n}\left( {{\mathbf{M}} - {\mathbf{J}}} \right)\left( {{\mathbf{M}} - {\mathbf{J}}} \right)^{\prime } + {\mathbf{11}}^{\prime }$ and because ${\mathbf{G}} = \frac{2}{n}\left( {{\mathbf{M}} - {\mathbf{J}}} \right)\left( {{\mathbf{M}} - {\mathbf{J}}} \right)^{\prime }$ it follows that ${\mathbf{G}}_{IBS} = \frac{1}{2}{\mathbf{G}} + {\mathbf{11}}^{\prime }$. The equivalence can also be verified by noting that, for all nine genotypes, the cross-product $\left( {m_{i} - 1} \right)\left( {m_{j} - 1} \right)$ in the following table is identical to $g_{IBS_{ij}} - 1$ in the previous table.

**Table:**

	AA	Aa	aa
AA	1	0	−1
Aa	0	0	0
Aa	−1	0	1

### Computation of the different ${\mathbf{H}}$ matrices

For ssGBLUP and ssGBLUP_F, matrix ${\mathbf{H}}^{ - 1}$ is constructed as follows [2, 3]:

$${\mathbf{H}}^{ - 1} = {\mathbf{A}}^{ - 1} + \left( {\begin{array}{*{20}c} {\mathbf{0}} & {\quad {\mathbf{0}}} \\ {\mathbf{0}} & {\quad {\mathbf{G}}_{a}^{* - 1} - {\mathbf{A}}_{22}^{ - 1} } \\ \end{array} } \right),$$

with ${\mathbf{G}}_{a}^{\varvec{*}} = 0.95{\mathbf{G}}_{a} + 0.05{\mathbf{A}}_{22} = 0.95\left( {a + b{\mathbf{G}}} \right) + 0.05{\mathbf{A}}_{22}$ and ${\mathbf{G}} = \frac{{\left( {{\mathbf{M}} - {\mathbf{P}}} \right)\left( {{\mathbf{M}} - {\mathbf{P}}} \right)}}{{2\sum p_{i} q_{i} }}$ as in [8], ${\mathbf{M}}$ contains genotypes coded as {0,1,2} and ${\mathbf{P}}$ contains twice allelic frequencies $p_{i}$. These are computed from the observed genotypes so that $2p_{i}$ is equal to the mean of the $i$-th column of ${\mathbf{M}}$. Constants $a$ and $b$ are such that the full-matrix and diagonal averages of ${\mathbf{G}}_{a}$ and ${\mathbf{A}}_{22}$ are the same [34] in order to make the two matrices compatible. The use of the weights 0.95 and 0.05 is in order to make ${\mathbf{G}}_{a}$ invertible. Matrix ${\mathbf{A}}^{ - 1}$ should be constructed using contributions with values described in the table below (i.e. [53]):

**Table:**

No parent known	1
One parent known	$\left( {0.75 - \frac{{F_{known} }}{4}} \right)^{ - 1}$
Two parents known	$\left( {0.5 - \frac{{F_{sire} }}{4} - \frac{{F_{dam} }}{4}} \right)^{ - 1}$

Or, in a more compact way $\left( {0.5 - \frac{{F_{sire} }}{4} - \frac{{F_{dam} }}{4}} \right)^{ - 1}$ with $F_{unknown} = - 1$.

ssGBLUP uses the defaults in blupf90 suite of programs (random_type *add_animal*). ssGBLUP uses the simple method to create ${\mathbf{A}}^{ - 1}$, a method which pretends that, in all cases, inbreeding in expressions above is $F = 0$.

ssGBLUP_F uses ${\mathbf{H}}^{ - 1}$ as above but constructs ${\mathbf{A}}^{ - 1}$ correctly (blupf90 random_type *add_an_upginb*), using the rules above.

ssGBLUP_M uses the blupf90 random_type *user_file* to consider the following relationship matrix, which was constructed externally:

$${\mathbf{H}}^{\left( \varGamma \right) - 1} = k\left( {{\mathbf{A}}^{\left( \varGamma \right) - 1} + \left( {\begin{array}{*{20}c} {\mathbf{0}} & {\mathbf{0}} \\ {\mathbf{0}} & {\quad {\mathbf{G}}^{{\varvec{*} - 1}} - {\mathbf{A}}_{22}^{\left( \varGamma \right) - 1} } \\ \end{array} } \right),} \right)$$

with ${\mathbf{G}}^{\varvec{*}} = 0.95{\mathbf{G}} + 0.05{\mathbf{A}}_{22}^{\left( \varGamma \right)}$ (basically to make $\varvec{G}$ invertible), ${\mathbf{G}} = \frac{1}{s}\left( {{\mathbf{M}} - {\mathbf{J}}} \right)\left( {{\mathbf{M}} - {\mathbf{J}}} \right)^{\prime }$ and $s = n/2$, ${\mathbf{M}}$ contains genotypes coded as {0,1,2}, $n$ is the number of markers, ${\mathbf{A}}^{\left( \varGamma \right) - 1}$ and ${\mathbf{A}}_{22}^{\left( \varGamma \right) - 1}$ are constructed with own programs as in [1] using the estimated value of ${\varvec{\Gamma}}$. Inbreeding is fully considered in both matrices ${\mathbf{A}}^{\left( \varGamma \right) - 1}$ and ${\mathbf{A}}_{22}^{\left( \varGamma \right) - 1}$. The constant $k = 1 - \frac{\gamma }{2}$ puts the genetic variance associated to metafounders (i.e. to “related” founders) on the same scale as regular “unrelated” founders in ${\mathbf{A}}$ or ${\mathbf{H}}$ [1].
